# Supplementary material for: Upper bounds for integrated information
Source: PLoS Comput Biol. 2024 Aug 5;20(8):e1012323. doi: 10.1371/journal.pcbi.1012323 (PMC11326638; doi:10.1371/journal.pcbi.1012323)
Supplement: S1 Appendix — Definition of the integrated cause information and description of how the results for integrated effect information can be translated to the integrated cause information. (PDF) [file pcbi.1012323.s001.pdf]

Upper bounds for integrated information

Alireza Zaeemzadeh[1*] and Giulio Tononi[1*]

1 Department of Psychiatry, University of Wisconsin, Madison, Wisconsin, United States of America.

* zaeemzadeh@wisc.edu (AZ), gtononi@wisc.edu (GT)

S1 - Integrated cause information

In Section 2.1, we discussed the definition and the upper bounds of the distinction integrated information. For simplicity, our discussion and theoretical results were mostly focused on the integrated effect information. Since $\varphi_d = \min\{\varphi_c, \varphi_e\} \leq \varphi_e$, any bound on φ_e or $\sum \varphi_e$ is a bound for φ_d or $\sum \varphi_d$. Here, for completeness, we discuss the definition of integrated cause information and show how the results obtained for the effect side can be translated to the cause side.

The cause repertoire $\pi_c(Z_{t-1} \mid M = m)$ is defined as as the probability distribution over a potential cause purview at time $t-1$, Z_{t-1}, given a mechanism in state m and can be calculated using Bayes' rule:

$$\pi_c(z \mid m) = \frac{\pi_e(m \mid z)\pi_c(z)}{\pi_e(m; Z)},$$

where $\pi_e(m; Z)$ is the unconstrained effect probability as defined in Section 2.1 and $\pi_c(z) = |\Omega_Z|^{-1}$ is the unconstrained cause probability. The time subscripts are dropped to avoid cluttering the notation. Similar to the effect side, the maximal cause state of the mechanism m over a potential cause purview Z can be found as:

$$z'_c(m, Z) = \arg\max_{z \in \Omega_Z} \pi_c(z \mid m) \log_2 \left(\frac{\pi_e(m \mid z)}{\pi_e(m; Z)} \right).$$

Since there is at least one state with $\pi_e(m \mid z) \geq \pi_e(m; Z)$, the maximal cause state is always a state for which the cause z increases the probability of the mechanism m compared to its unconstrained probability. Finally, the integrated cause information is

calculated as:

$$\varphi_c(m, Z) = \pi_c(z'_c \mid m) \left| \log_2 \left(\frac{\pi_e(m \mid z'_c)}{\pi_e^{\theta'}(m \mid z'_c)} \right) \right|_+ , \tag{17}$$

where θ' is the minimum information partition (MIP), the partition that achieves the minimum normalized integrated cause information. We can also find the most irreducible cause purview of a mechanism as:

$$z_c^*(m) = \arg \max_{\{z'_c \mid Z \subseteq S\}} \varphi_c(m, Z = z'_c).$$

These steps are similar to the steps described for the effect side in Section 2.1. The only difference is the definition of the repertoires and the use of a slightly different distance measure. The measure being used for the cause side has the form $\pi_c(z \mid m) \left| \log_2 \left(\frac{\pi_e(m|z)}{\pi'_e(m|z)} \right) \right|_+$, where the selectivity uses the backward probability and the informativeness uses the forward probability. This measure satisfies the following properties: (i) The measure differs from 0 only if the cause state increases the probability of the mechanism state. (ii) The measure is not an aggregate over all the states and reflects how much change is made in an individual state. (iii) In a scenario where adding more units to the cause purview does not increase the probability of the mechanism state further, having the new units in the maximally irreducible purview is discouraged.

More importantly, since the informativeness term is the same for both φ_e and φ_c, all the results described for φ_e hold for φ_c as well. For example, the normalization factor derived in Lemma 2 holds for both the cause and effect information, as it is a bound on the infromativeness term. Similarly, since the proofs for Theorem 2 and Theorem 3 only use the informativeness term to derive a bound for the integrated effect information, they both hold for the integrated cause information.
