# Supplementary material for: Upper bounds for integrated information
Source: PLoS Comput Biol. 2024 Aug 5;20(8):e1012323. doi: 10.1371/journal.pcbi.1012323 (PMC11326638; doi:10.1371/journal.pcbi.1012323)
Supplement: S2 Appendix — Describes how the results change if other difference measures, such as KL divergence, are used for comparing the distributions. (PDF) [file pcbi.1012323.s002.pdf]

Upper bounds for integrated information

Alireza Zaeemzadeh[1*] and Giulio Tononi[1*]

1 Department of Psychiatry, University of Wisconsin, Madison, Wisconsin, United States of America.

* zaeemzadeh@wisc.edu (AZ), gtononi@wisc.edu (GT)

S2 - Using other difference measures

As discussed in Section 2.1 and S1 Appendix, the difference measure to quantify φ_e and φ_c satisfy certain properties. In this section, we study how our results might change if we change the difference measure to satisfy slightly different properties.

1 Absolute informativeness

Due to using the positive part operator $|.|_+$ in the informativeness, integrated information is non-zero only if the state at $t-1$ increases the probability of the state at t. This matches our intuition that a cause should increase the probability of its effect. However, we can show even if we use the absolute value operator in the difference measure, our results would stay the same, by showing that the upper bound for the negative case is smaller than the upper bound for the positive case. Lemma 1 states the upper bound for the case where the mechanism *decreases* the probability.

Lemma 1 (Maximum for negative cause/effect)**.** *For* $0 \leq p, q \leq 1$*, if* $p \leq q$ *then*

$$p\left|\log_2(\frac{p}{q})\right| \leq q\frac{\log_2(e)}{e} \leq \frac{\log_2(e)}{e} \approx 0.531.$$

Proof. For $0 \le p \le q \le 1$, we have $p \left| \log_2(\frac{p}{q}) \right| = -p \log_2(\frac{p}{q})$ and

$$\frac{d(-p \log_2(\frac{p}{q}))}{dp} = \frac{-\ln(\frac{p}{q}) - 1}{\ln(2)}.$$
$$\frac{d^2(-p \log_2(\frac{p}{q}))}{dp^2} = -\frac{1}{\ln(2)p} < 0.$$

Therefore, $p \left| \log_2(\frac{p}{q}) \right|$ is concave for $0 \le p \le q \le 1$. The maximum is obtained by setting the first derivative to 0:

$$\frac{d(-p \log_2(\frac{p}{q}))}{dp} = \frac{-\ln(\frac{p}{q}) - 1}{\ln(2)} = 0$$
$$p = \frac{q}{e}$$

For $p = \frac{q}{e}$, we have $p \left| \log_2(\frac{p}{q}) \right| = \frac{q}{e} \log_2(e) \le \frac{\log_2(e)}{e}$. Thus for any $p \le q$, $p \left| \log_2(\frac{p}{q}) \right| \le \frac{\log_2(e)}{e}$. □

In other words, achieving integrated information of larger than $\frac{\log_2(e)}{e} \approx 0.531$ is not possible by decreasing the probability. Therefore, the mechanism needs to increase the probability to achieve large values of integrated information. All the bounds discussed in Section 2.1 are larger than this value, therefore changing $|.|_+$ to $|.|$ does not change our results.

2 Point-wise mutual information

Another candidate for the distance measure is the point-wise mutual information, *i.e.,* $\left| \log(\frac{p}{q}) \right|_+$. Similar to the measure used in the definition of the integrated information, this measure is not an aggregate over the states and quantifies the change in an individual state. Furthermore, it differs from 0 only if $p > q$. This measure has been used previously to quantify actual causation is discrete systems [1]. Using this measure does not change our results because the bounds derived in Section 2.1 were all derived for the informativeness term of difference measure, which has the form $\left| \log(\frac{p}{q}) \right|_+$.

3 Kullback–Leibler divergence

Kullback–Leibler divergence (KLD) measure is another common distance measure for probability distribution and is defined as $D_{KL}(p(X)||q(X)) = \sum_{x\in\Omega_X} p(X=x)\log(\frac{p(X=x)}{q(X=x)})$. It is 0, only if $p(X=x) = q(X=x), \forall x \in \Omega_X$, and is an aggregate measure over all the states, unlike our default measure.

Here, we study the upper bound for $D_{KL}(\pi_e(Z \mid m)||\pi_e^\theta(Z \mid m))$ and show that the results in Section 2.1 are still relevant even if we use KLD. First, using the definition of the effect repertoire and additivity of KLD, we have:

$$D_{KL}(\pi_e(Z \mid m)||\pi_e^\theta(Z \mid m)) = D_{KL}(\prod_{Z_i\in Z} \pi_e(Z_i \mid m)|| \prod_{Z_i\in Z} \pi_e^\theta(Z_i \mid m)) = \sum_{Z_i\in Z} D_{KL}(\pi_e(Z_i \mid m)||\pi_e^\theta(Z_i \mid m)). \quad (1)$$

Let us define the mechanism connected to Z_i after the partitioning as M_i. For each individual binary unit in the purview Z_i, we can use the proof in lemma 1 to show that:

$$\frac{\pi_e(Z_i = z_i \mid m)}{\pi_e(Z_i = z_i \mid m_i)} \leq 2^{|M|-|M_i|}.$$

Therefore, finding the maximum value for an individual binary unit boils down to solving the following problem:

$$\begin{aligned} \max_{p,q} \quad & D_{KL}(p||q) = p\log(\frac{p}{q}) + (1-p)\log(\frac{1-p}{1-q}) \\ \text{subject to} \quad & p \leq 2^{|M|-|M_i|}q \\ & q \leq p \leq 1. \end{aligned}$$

The constraint $q \leq p$ is added without loss of generality, as it always holds for one of the two states. We are denoting the probabilities of that state with p and q. Under this constraint the derivative of the objective function with respect to p is always non-negative:

$$\frac{\partial D_{KL}(p||q)}{\partial p} = \log\left(\frac{p}{q}\right) - \log\left(\frac{1-p}{1-q}\right) = \log\left(\frac{\frac{1}{q}-1}{\frac{1}{p}-1}\right) \geq 0$$

This means the maximum of $D_{KL}(p||q)$ occurs at the boundary for which p is the

largest, which is $p = \min\{1, 2^{|M|-|M_i|}q\}$. In other words, $p = 2^{|M|-|M_i|}q$ for $q \leq \frac{1}{2}^{|M|-|M_i|}$ and $p = 1$, otherwise. For the latter case, we have $D_{KL}(p||q) = \log(\frac{1}{q})$, whose maximum occurs when q is minimized, *i.e.,* $q = \frac{1}{2}^{|M|-|M_i|}$, with the maximal KLD value of $|M| - |M_i|$. The maximum for the former case also happens at the same point, since the derivative of the objective function with respect to q is non-negative:

$$\frac{\partial}{\partial q}\left(2^{|M|-|M_i|}q(|M| - |M_i|) + (1 - 2^{|M|-|M_i|}q)\log(\frac{1 - 2^{|M|-|M_i|}q}{1-q})\right) \geq 0,$$

and we need to maximize q in order to maximize $D_{KL}(p||q)$. This again leads to the maximum value of $|M| - |M_i|$ at $q = \frac{1}{2}^{|M|-|M_i|}$. Thus, we have:

$$D_{KL}(\pi_e(Z_i \mid m)||\pi_e(Z_i \mid m_i)) \leq |M| - |M_i|,$$

which is the same bound as Lemma 1, but for KLD instead of our default distance measure. $|M| - |M_i|$ is the number of connection severed form Z_i. We can plug this result into (1) and arrive at:

$$D_{KL}(\pi_e(Z \mid m)||\pi_e^{\theta}(Z \mid m)) = \sum_{Z_i \in Z} D_{KL}(\pi_e(Z_i \mid m)||\pi_e^{\theta}(Z_i \mid m)) \leq \sum_{Z_i \in Z}(|M| - |M_i|) = \mathcal{N}(\theta).$$

$\mathcal{N}(\theta)$ is the number of connections severed by the partition θ. This is the same result as Lemma 2 and shows that even if we use KLD as the distance measure, the normalization factor for finding the MIP would not change. This can also be used to show that if we use KLD to quantify φ_e, it cannot be larger than the number of connections between the mechanism and the purview, *i.e.,* $\varphi_e(m, E) \leq |M||E|$ (same results as Theorem 1).

Furthermore, since KLD is maximized when $\pi_e(Z \mid m)$ is fully deterministic, Lemma 4 and Theorem 2 hold. Finally, the assumption for Theorem 3 is that the effect repertoire is fully deterministic, *i.e.,* $\pi_e(Z = z \mid m) = 1$ for some $z \in \Omega_Z$. In this case, KLD is simplified to $\log(\frac{1}{\pi_e^{\theta}(Z=z|m)}) = \pi_e(Z = z \mid m)\left|\log(\frac{\pi_e(Z=z|m)}{\pi_e^{\theta}(Z=z|m)})\right|_+$, which coincides with our default distance measure. Therefore, Theorem 3 holds as well.

References

1. Albantakis L, Marshall W, Hoel E, Tononi G. What Caused What? A Quantitative Account of Actual Causation Using Dynamical Causal Networks. Entropy 2019, Vol 21, Page 459. 2019;21(5):459. doi:10.3390/E21050459.
