# Supplementary material for: Upper bounds for integrated information
Source: PLoS Comput Biol. 2024 Aug 5;20(8):e1012323. doi: 10.1371/journal.pcbi.1012323 (PMC11326638; doi:10.1371/journal.pcbi.1012323)
Supplement: S3 Appendix — (PDF) [file pcbi.1012323.s003.pdf]

Upper bounds for integrated information

Alireza Zaeemzadeh[1*] and Giulio Tononi[1*]

1 Department of Psychiatry, University of Wisconsin, Madison, Wisconsin, United States of America.

* zaeemzadeh@wisc.edu (AZ), gtononi@wisc.edu (GT)

S3 - Proofs

1 Single mechanism

Before presenting our main proofs, let us revisit the definition of single-unit effect repertoire. Given the set of units outside the mechanism $W = S - M$ and a single unit Z_i in state z_i:

$$\pi_e(Z_i = z_i \mid m) = \frac{1}{2^{|W|}} \sum_w p(Z_i = z_i \mid m, w) = \frac{1}{|\mathcal{M}(m)|} \sum_{s \in \mathcal{M}(m)} p(Z_i = z_i \mid s),$$

where $\mathcal{M}(m) \subset \Omega_S$ is the set of system states in which mechanism M is in state m and we have $|\mathcal{M}(m)| = 2^{|W|} = 2^{N-|M|}$. Ω_S is the set of all possible states of the system S. Defining $\mathcal{M}(m)$ helps us to present our results in a more concise manner. For example, we will use the following simple lemma to prove Lemma 1

Lemma 5. *For two mechanisms M and $\bar{M}$ such that $\bar{M} \subset M \subseteq S$, we have $\mathcal{M}(m) \subset \mathcal{M}(\bar{m})$.*

Proof. Without loss of generality let us assume M is in all-zero state. By definition, $\forall s \in \mathcal{M}(m)$ the units corresponding to M are in all-zero state. Since $\bar{M} \subset M$, $\forall s \in \mathcal{M}(m)$ the units corresponding to $\bar{M}$ are also in all-zero state and therefore $s \in \mathcal{M}(\bar{m})$. Thus, $\forall s \in \mathcal{M}(m)$, we have $s \in \mathcal{M}(\bar{m})$, which means $\mathcal{M}(m) \subset \mathcal{M}(\bar{m})$. □

Example 1. *$\bar{M} = A$ and $M = AB$ for system $S = ABC$ in all-zero state.*
$\mathcal{M}(\bar{m}) = \{ABC = 000, ABC = 001, ABC = 010, ABC = 011\}$ and
$\mathcal{M}(m) = \{ABC = 000, ABC = 001\}$.

Lemma 1. *Given two mechanisms $\bar{M}$ and M, such that $\bar{M} \subset M \subseteq S$, and a single unit Z_i, we have:*

$$\left| \log_2\left(\frac{\pi_e(Z_i = z_i \mid m)}{\pi_e(Z_i = z_i \mid \bar{m})}\right) \right|_+ \leq |M| - |\bar{M}|.$$

Proof. For a single purview unit $Z_i \in Z$ we have:

$$\frac{\pi_e(Z_i = z_i \mid m)}{\pi_e(Z_i = z_i \mid \bar{m})} = \frac{\frac{1}{|\mathcal{M}(m)|} \sum_{s \in \mathcal{M}(m)} p(Z_i = z_i \mid s)}{\frac{1}{|\mathcal{M}(\bar{m})|} \sum_{s \in \mathcal{M}(\bar{m})} p(Z_i = z_i \mid s)}$$

Since $\bar{M} \subset M$, we have $\mathcal{M}(m) \subset \mathcal{M}(\bar{m})$ (using Lemma 5), therefore $\sum_{s \in \mathcal{M}(m)} p(Z_i = z_i \mid s) \leq \sum_{s \in \mathcal{M}(\bar{m})} p(Z_i = z_i \mid s)$. This is because we are taking the sum over more terms on the right side. Thus

$$\frac{\pi_e(Z_i = z_i \mid m)}{\pi_e(Z_i = z_i \mid \bar{m})} \leq \frac{\frac{1}{|\mathcal{M}(m)|}}{\frac{1}{|\mathcal{M}(\bar{m})|}} = \frac{\frac{1}{2^{N-|M|}}}{\frac{1}{2^{N-|\bar{M}|}}} = 2^{|M|-|\bar{M}|}.$$

□

Lemma 2. *Given a mechanism M in state m, a purview Z in state z, and a partition θ, we have:*

$$\left| \log_2\left(\frac{\pi_e(Z = z \mid m)}{\pi_e^\theta(Z = z \mid m)}\right) \right|_+ \leq \mathcal{N}(\theta),$$

where $\mathcal{N}(\theta)$ is the total number of connections cut by the partition θ.

Proof. Due to the conditional independence of the purview units, we have:

$$\log_2\left(\frac{\pi_e(Z = z \mid m)}{\pi_e^\theta(Z = z \mid m)}\right) = \log_2\left(\frac{\prod_i \pi_e(Z_i = z_i \mid m)}{\prod_i \pi_e^\theta(Z_i = z_i \mid m)}\right) = \sum_{Z_i \in Z} \log_2\left(\frac{\pi_e(Z_i = z_i \mid m)}{\pi_e^\theta(Z_i = z_i \mid m)}\right)$$

Let us define M_i as the mechanism connected to Z_i after the partitioning. Using Lemma 1:

$$\sum_{Z_i \in Z} \log_2\left(\frac{\pi_e(Z_i = z_i \mid m)}{\pi_e^\theta(Z_i = z_i \mid m)}\right) \leq \sum_{Z_i \in Z} (|M| - |M_i|) = \mathcal{N}(\theta).$$

$\mathcal{N}(\theta) = \sum_{Z_i \in Z}(|M| - |M_i|)$ is the total number of connections cut by partition θ. □

Theorem 1. *For a mechanism $M \subseteq S$ in state m, a candidate cause purview C, and a candidate effect purview E, we have:*

$$\varphi_e(m, E) \leq |M||E| \quad \text{and} \quad \varphi_c(m, C) \leq |M||C|,$$

where $|E|$ and $|C|$ denote the size of the candidate effect and cause purviews, respectively.

Proof. For the effect side, we have:

$$\varphi_e(m, E) = \pi_e(E = e \mid m) \left| \log_2\left(\frac{\pi_e(E = e \mid m)}{\pi_e^{\theta'}(E = e \mid m)}\right) \right|_+ \leq \left| \log_2\left(\frac{\pi_e(E = e \mid m)}{\pi_e^{\theta'}(E = e \mid m)}\right) \right|_+$$
$$\leq \max_\theta \left| \log_2\left(\frac{\pi_e(E = e \mid m)}{\pi_e^{\theta}(E = e \mid m)}\right) \right|_+ \overset{(a)}{\leq} \max_\theta \mathcal{N}(\theta) \leq |M||E|$$

θ' represents the MIP and equality (a) follows from Lemma 2. Similarly for the cause side, we have:

$$\varphi_c(m, C) = \pi_c(C = c \mid m) \left| \log_2\left(\frac{\pi_e(M = m \mid c)}{\pi_e^{\theta'}(M = m \mid c)}\right) \right|_+ \leq \left| \log_2\left(\frac{\pi_e(M = m \mid c)}{\pi_e^{\theta'}(M = m \mid c)}\right) \right|_+$$
$$\leq \max_\theta \left| \log_2\left(\frac{\pi_e(M = m \mid c)}{\pi_e^{\theta}(M = m \mid c)}\right) \right|_+ \leq \max_\theta \mathcal{N}(\theta) \leq |M||C|$$

□

Lemma 4. *If $\varphi_e(m, Z) = |M||Z|$ then $\pi_e(Z = z \mid m) = 1$. Similarly, if $\varphi_c(m, Z) = |M||Z|$ then $\pi_c(Z = z \mid m) = 1$.*

Proof. In deriving the upper bounds in Theorem 1, the upper bound is achieved by setting $\pi_e(E = e \mid m)$ and $\pi_c(C = c \mid m)$ to 1. Using the same line of proof and setting $\pi_e(E = e \mid m) < 1$ and $\pi_c(C = c \mid m) < 1$, we will achieve $\varphi_e(m, E) < |M||E|$ and $\varphi_c(m, C) < |M||C|$. Thus, to achieve $\varphi_e(m, E) = |M||E|$ ($\varphi_c(m, C) = |M||C|$), we need to have $\pi_e(E = e \mid m) = 1$ ($\pi_c(C = c \mid m) = 1$). □

2 Inter-order constraints

To prove Theroem 2, we first need to prove a few intermediate lemmas.

Lemma 6 (Deterministic mechanism)**.** *For a mechanism $M \subseteq S$ and a single-unit purview Z_i, if $\pi_e(Z_i = z_i \mid m) = 1$, then $p(Z_i = z_i \mid s) = 1, \forall s \in \mathcal{M}(m)$. Furthermore, if $\pi_e(Z_i = z_i \mid m) = 0$, then $p(Z_i = z_i \mid s) = 0, \forall s \in \mathcal{M}(m)$.*

Proof. For $\pi_e(Z_i = z_i \mid m) = 1$:

$$\pi_e(Z_i = z_i \mid m) = \frac{1}{|\mathcal{M}(m)|} \sum_{s \in \mathcal{M}(m)} p(Z_i = z_i \mid s) = 1.$$

Therefore, $\sum_{s \in \mathcal{M}(m)} p(Z_i = z_i \mid s) = |\mathcal{M}(m)|$. Since $p(Z_i = z_i \mid s) \leq 1, \forall s$, all the terms in the summation need to be 1, *i.e.*, $p(Z_i = z_i \mid s) = 1, \forall s \in \mathcal{M}(m)$.

For $\pi_e(Z_i = z_i \mid m) = 0$, $\sum_{s \in \mathcal{M}(m)} p(Z_i = z_i \mid s) = 0$. Since $p(Z_i = z_i \mid s) \geq 0, \forall s$, all the terms in the summation need to be 0, *i.e.*, $p(Z_i = z_i \mid s) = 0, \forall s \in \mathcal{M}(m)$. □

Lemma 3. *Superset of a deterministic mechanism is deterministic. For two mechanisms $M \subset \bar{M} \subseteq S$ and a single-unit purview Z_i, If $\pi_e(Z_i = z_i \mid m) = 1$, then $\pi_e(Z_i = z_i \mid \bar{m}) = 1$ and if $\pi_e(Z_i = z_i \mid m) = 0$, then $\pi_e(Z_i = z_i \mid \bar{m}) = 0$.*

Proof. If $\pi_e(Z_i = z_i \mid m) = 1$:

According to Lemma 6, $p(Z_i = z_i \mid s) = 1, \forall s \in \mathcal{M}(m)$. Since $M \subset \bar{M}$, we have $\mathcal{M}(\bar{m}) \subset \mathcal{M}(m)$ (Lemma 5). Therefore, $p(Z_i = z_i \mid s) = 1, \forall s \in \mathcal{M}(\bar{m})$ and $\pi_e(Z_i = z_i \mid \bar{m}) = \frac{1}{|\mathcal{M}(\bar{m})|} \sum_{s \in \mathcal{M}(\bar{m})} p(Z_i = z_i \mid s) = 1$.

Similarly, for $\pi_e(Z_i = z_i \mid m) = 0$:

According to Lemma 6, $p(Z_i = z_i \mid s) = 0, \forall s \in \mathcal{M}(m)$. Since $M \subset \bar{M}$, we have $\mathcal{M}(\bar{m}) \subset \mathcal{M}(m)$ (Lemma 5). Therefore, $p(Z_i = z_i \mid s) = 0, \forall s \in \mathcal{M}(\bar{m})$ and $\pi_e(Z_i = z_i \mid \bar{m}) = \frac{1}{|\mathcal{M}(\bar{m})|} \sum_{s \in \mathcal{M}(\bar{m})} p(Z_i = z_i \mid s) = 0$. □

Lemma 7. *If $\varphi_e(m, Z) = |M||Z|$ then $\varphi_e(\bar{m}, \bar{Z}) < |\bar{M}||\bar{Z}|$, if $M \subset \bar{M}$ and $Z \cap \bar{Z} \neq \varnothing$.*

Proof. Since $\varphi_e(m, Z) = |M||Z|$, using Lemma 4:

$$\begin{aligned}
&\pi_e(Z = z^* \mid m) = \prod_{Z_i \in Z} \pi_e(Z_i = z_i^* \mid m) = 1 \\
&\Longrightarrow \pi_e(Z_i = z_i^* \mid m) = 1, \forall Z_i \in Z \\
&\overset{(a)}{\Longrightarrow} p(Z_i = z_i \mid s) = 1, \forall s \in \mathcal{M}(m), \forall Z_i \in Z \\
&\Longrightarrow \pi_e(Z_i = z_i^*) = \frac{1}{|\Omega_S|} \sum_{s \in \Omega_S} p(Z_i = z_i \mid s) \geq \frac{|\mathcal{M}(m)|}{|\Omega_S|} = \frac{2^{N-|M|}}{2^N} = \frac{1}{2^{|M|}}, \forall Z_i \in Z,
\end{aligned}$$

From Theorem 1, we already know $\varphi_e(\bar{m}, \bar{Z}) \leq |\bar{M}||\bar{Z}|$ and from Lemma 2, we know the only partitioning that can achieve $|\bar{M}||\bar{Z}|$ is the complete partition, *i.e.*, removing all the connections between $\bar{M}$ and $\bar{Z}$. Now, we show even the complete partition cannot achieve $|\bar{M}||\bar{Z}|$. Under the complete partition, $\theta = \{(\varnothing, \bar{M}), (\bar{Z}, \varnothing)\}$, we have:

$$\varphi_e(\bar{m},\bar{Z},\theta)=\pi_e(\bar{Z}=\bar{z}\mid\bar{m})\left|\log_2(\frac{\pi_e(\bar{Z}=\bar{z}\mid\bar{m})}{\pi_e(\bar{Z}=\bar{z})})\right|_+\leq\left|\log_2(\frac{\pi_e(\bar{Z}=\bar{z}\mid\bar{m})}{\pi_e(\bar{Z}=\bar{z})})\right|_+$$
$$=\left|\sum_{Z_i\in\bar{Z}}\log_2(\frac{\pi_e(\bar{Z}_i=\bar{z}_i\mid\bar{m})}{\pi_e(\bar{Z}_i=\bar{z}_i)})\right|_+\leq\left|\sum_{Z_i\in\bar{Z}\cap Z}\log_2(\frac{\pi_e(\bar{Z}_i=\bar{z}_i\mid\bar{m})}{\pi_e(\bar{Z}_i=\bar{z}_i)})\right|_++\left|\sum_{Z_i\in\bar{Z}-Z}\log_2(\frac{\pi_e(\bar{Z}_i=\bar{z}_i\mid\bar{m})}{\pi_e(\bar{Z}_i=\bar{z}_i)})\right|_+$$
$$\stackrel{(a)}{\leq}|\bar{Z}\cap Z||M|+|\bar{Z}-Z||\bar{M}|<|\bar{Z}\cap Z||\bar{M}|+|\bar{Z}-Z||\bar{M}|=|\bar{Z}||\bar{M}|$$

The first term in (a) follows from the fact that $\pi_e(Z_i=z_i^*)\geq\frac{1}{2^{|M|}},\forall Z_i\in Z$, as we proved earlier, and the second term follows from Lemma 1. □

Lemma 8. *$\varphi_e(\bar{m},\bar{Z})<|\bar{M}||\bar{Z}|$, if $\varphi_e(m,Z)=|M||Z|$ and $\bar{M}\subset M$ and $Z\cap\bar{Z}\neq\varnothing$.*

Proof. Proof by contradiction: Assume $\varphi(\bar{m},\bar{Z})=|\bar{M}||\bar{Z}|$. Then according to Lemma 7, $\varphi(m,Z)<|M||Z|$. Contradiction. □

Theorem 2. *$\varphi_e(\bar{m},\bar{Z})<|\bar{M}||\bar{Z}|$, if $\varphi_e(m,Z)=|M||Z|$ and*

- *$\bar{M}\subset M$ and $Z\cap\bar{Z}\neq\varnothing$, OR*
- *$\bar{M}\supset M$ and $Z\cap\bar{Z}\neq\varnothing$.*

Proof. Follows directly from Lemma 8 and Lemma 7. □

3 Intra-order constraints

In this section, we study a system consisting of N units in which all the mechanisms of size K specify themselves with probability 1:

$$\pi_e(Z=z'\mid m)=1,\forall M:|M|=K,Z=M.$$

First, we show how we can construct a TPM that can satisfy this property. Then, we show why such mechanisms cannot achieve their maximal value. Finally, we show, for such mechanism, MIP can only be one of a few candidate partitions.

Let us denote $M_{n,j}$ and $Z_{n,j}$, $j=1,\ldots,\binom{N-1}{K-1}$ and $n=1,\ldots,N$ as the set of mechanisms and purviews of size K that contain n^{th} unit, respectively. From the assumptions of the theorem we also have $Z_{n,j}=M_{n,j},|Z_{n,j}|=|M_{n,j}|=K,\forall j,n$. Furthermore, Z_n denotes the single-unit purview only containing the nth unit. Starting

from the assumption of the theorem, we have:

$$\pi_e(Z_{n,j} = z^*_{n,j} \mid m_{n,j}) = 1, \forall n, j \implies \pi_e(Z_n = z^*_n \mid m_{n,j}) \quad = 1, \forall n, j$$

This expression means the probability of the nth purview unit, given all the mechanisms of size K that contain the nth unit is 1. Using Lemma 6:

$$p(Z_n = z^*_n \mid s) = 1, \forall s \in \mathcal{M}(m_{n,j}), n = 1, \ldots, N, \forall j$$

Note that in general z^*_n, which is the maximal state the purview unit, depends on the mechanism $M_{n,j}$. But the above constraint is only satisfiable when z^*_n is the same for all j. To see that, first notice that $\mathcal{M}(m_{n,j}) \cap \mathcal{M}(m_{n,j'}) \neq \varnothing, \forall j, j'$ and for $s \in \mathcal{M}(m_{n,j}) \cap \mathcal{M}(m_{n,j'})$, either we have $p(Z_n = 0 \mid s) = 1$ or $p(Z_n = 1 \mid s) = 1$. Therefore, $M_{n,j}$ and $M_{n,j'}$ should agree on the state of Z_n. This is true for any pair j, j'. Without loss of generality, we assume z^*_n is 0 for all n and j.

$$p(Z_n = 0 \mid s) = 1, \forall s \in \bigcup_j \mathcal{M}(m_{n,j}), n = 1, \ldots, N. \tag{18}$$

Furthermore, without loss of generality, let us assume that the system is also in all-zero state at the current time. Thus, $\mathcal{M}(m_{n,j})$ is the set of system states in which the state of the units in $M_{n,j}$ (unit n and $K-1$ other units) are 0 and $\bigcup_j \mathcal{M}(m_{n,j})$ is the set of all the system states for which the state of the unit n and at least $K-1$ other units are 0.

So far, we have shown that, for any single purview unit Z_n, the TPM entry for $p(Z_n = 0 \mid s)$ is 1 for all system states s that the unit n and at least $K-1$ other units are 0. To study the partitioned repertoires, we should also derive $\pi_e(Z_n = 0 \mid \bar{m})$ for different mechanisms $\bar{m} \subset m_{n,j}$ with $|\bar{M}| < K$.

First, consider the case where $\bar{M}$ contains unit n and $|\bar{M}| < K$:

$$\pi_e(Z_n = 0 \mid \bar{m} = 0) = \frac{1}{|\mathcal{M}(\bar{m})|} \sum_{s \in \mathcal{M}(\bar{m})} p(Z_n = 0 \mid s),$$

Let us denote this probability as $\pi(|\bar{M}|)$. $\mathcal{M}(\bar{m})$ is the set of system states for which unit n and $|\bar{M}|-1$ other units are 0 and we are marginalizing over the state of the rest

of $N - |\bar{M}|$ units. But as we discussed earlier, we know any state with unit n and at least $K-1$ other units in state 0 has $p(Z_n = 0 \mid s) = 1$. So out of the $2^{N-|\bar{M}|}$ states in $\mathcal{M}(\bar{m})$, there are at least $\sum_{b=K-|\bar{M}|}^{N-|\bar{M}|} \binom{N-|\bar{M}|}{b}$ states with probability 1. Therefore:

$$\pi(|\bar{M}|) \geq \frac{\sum_{b=K-|\bar{M}|}^{N-|\bar{M}|} \binom{N-|\bar{M}|}{b}}{2^{N-|\bar{M}|}}.$$

Similarly, we can define the probability $\pi_e(Z_n = 0 \mid \bar{m} = 0)$ for the case where $\bar{M}$ does not contain unit n and $|\bar{M}| < K$ as $\bar{\pi}(|\bar{M}|)$. In this case, $\mathcal{M}(\bar{m})$ is the set of system states for which $|\bar{M}|$ units are 0 and we are marginalizing over the state of the rest of $N - |\bar{M}|$ units. Again, any state in $\mathcal{M}(\bar{m})$ with unit n and $K - |\bar{M}| - 1$ other units in state 0 has probability of 1. Therefore:

$$\bar{\pi}(|\bar{M}|) \geq \frac{\sum_{b=K-|\bar{M}|-1}^{N-|\bar{M}|-1} \binom{N-|\bar{M}|-1}{b}}{2^{N-|\bar{M}|}} = \frac{\sum_{b=K-|\bar{M}|}^{N-|\bar{M}|} \binom{N-|\bar{M}|-1}{b-1}}{2^{N-|\bar{M}|}}.$$

To find the MIP, we need to find the minimum normalized difference between partitioned and unpartitioned repertoires for the pair Z and M:

$$\frac{1}{\mathcal{N}(\theta)} \pi_e(Z = z \mid m) \left| \log_2\left(\frac{\pi_e(Z = z \mid m)}{\pi_e^\theta(Z = z \mid m)}\right) \right|_+ = \frac{1}{\mathcal{N}(\theta)} \log_2\left(\frac{1}{\prod_i \pi_e^\theta(Z_i = z_i \mid m)}\right) = -\frac{1}{\mathcal{N}(\theta)} \sum_{Z_i \in Z} \log_2(\pi_e(Z_i = z_i \mid m_i)) \quad (19)$$

m_i is the mechanism connected to Z_i after the partition and $\pi_e^\theta(Z_i = z_i \mid m_i)$ is either $\pi(|M_i|)$ and $\bar{\pi}(|M_i|))$. The lower bounds for both $\pi(|\bar{M}|)$ and $\bar{\pi}(|\bar{M}|))$, therefore the upper bound for the sum, are achieved when:

$$p(Z_n = 0 \mid s) = 0, \forall s \notin \bigcup_j \mathcal{M}(m_{n,j}), n = 1, \ldots, N. \quad (20)$$

Eq (18) and Eq (20) provide us with $p(Z_n = 0 \mid s)$ for all system states s and purview units n, which gives us the full TPM.

We can even further decompose the sum in Eq (19) into sum over individual connections. Assume we are removing connections from the mechanism m to the purview unit z_i one by one until we arrive at the mechanism after the partitioning m_i. Let us denote the intermediate steps as $m_i^{(0)}, m_i^{(1)}, m_i^{(2)}, \ldots, m_i^{(N_i)}$, where $m_i^{(0)} = m$

and $m_i^{(N_i)} = m_i$ and N_i is the number of connections cut from z_i. We can write the normalized partitioned informativeness as:

$$\frac{1}{\mathcal{N}(\theta)} \left| \sum_{Z_i \in Z} \sum_{c=1}^{N_i} \log_2\left(\frac{\pi_e(Z = z \mid m_i^{(c-1)})}{\pi_e(Z = z \mid m_i^{(c)})}\right) \right|_+ = \frac{1}{\mathcal{N}(\theta)} \sum_{Z_i \in Z} \sum_{c=1}^{N_i} \log_2\left(\frac{\pi_e(Z = z \mid m_i^{(c-1)})}{\pi_e(Z = z \mid m_i^{(c)})}\right) \quad (21)$$

Positive part operator can be removed as both $\pi(|\bar{M}|)$ and $\bar{\pi}(|\bar{M}|))$ decrease as the size of the mechanism $|\bar{M}|$ increases, making all the terms inside the sum positive. Eq (21) rewrites the normalized partitioned informativeness as the average informativeness gain over the individual connections severed by the partition.

From Lemma 1, we know the information gain per connection can at most be 1. Now, using $\pi(|\bar{M}|)$ and $\bar{\pi}(|\bar{M}|))$, we can make a few observations about the information gain of removing different connections in the system under consideration. First, we show cutting a self connection can achieve the maximum information gain of 1. This means removing the self connection cannot decrease the normalized partitioned informativeness, as adding 1 to a set of numbers that can at most be 1 cannot decrease the average. To calculate the information gain for cutting a self connection, we should compare $\pi(|M_i|)$ to $\bar{\pi}(|M_i| - 1)$, *i.e.,* cutting one input connection from unit Z_i such that the mechanism connected to it no longer contains it:

$$\begin{aligned} \pi(|M_i|)) &= \frac{\sum_{b=K-|M_i|}^{N-|M_i|} \binom{N-|M_i|}{b}}{2^{N-|M_i|}} = \frac{2\sum_{b=K-|M_i|}^{N-|M_i|} \binom{N-|M_i|}{b}}{2^{N-|M_i|+1}} \\ &= 2\frac{\sum_{b=K-(|M_i|-1)-1}^{N-(|M_i|-1)-1} \binom{N-(|M_i|-1)-1}{b}}{2^{N-(|M_i|-1)}} = 2\bar{\pi}(|M_i| - 1)). \end{aligned}$$

This provides us with the proof for Theorem 3.

Theorem 3. *In a system S consisting of N units, for a given mechanism size $1 < K < N$, if $\pi_e(Z = z' \mid m) = 1, \forall M : |M| = K$, and the purview units are the same as the mechanism units,* i.e., *$Z = M$, none of the mechanisms with $|M| = K$ can achieve their maximum integrated effect information of $|M||Z| = |M|^2$.*

Proof. Achieving the maximum value is only possible if the MIP is the complete partition. Since, as shown above, not having the self connections in the cut for $1 < K < N$ gives us a smaller normalized partitioned informativeness, the complete partition cannot be the MIP. □

We can similarly calculate the informativeness gain for a lateral connection as

$\log_2(\frac{\pi(|\bar{M}|)}{\pi(|\bar{M}|-1)})$ or $\log_2(\frac{\bar{\pi}(|\bar{M}|)}{\bar{\pi}(|\bar{M}|-1)})$, depending on if the self connection is intact or not, respectively. In the first case we have:

$$\log_2(\frac{\pi(|\bar{M}|)}{\pi(|\bar{M}|-1)}) = \log_2(2\frac{\sum_{b=K-|\bar{M}|}^{N-|\bar{M}|}\binom{N-|\bar{M}|}{b})}{\sum_{b=K-|\bar{M}|+1}^{N-|\bar{M}|+1}\binom{N-|\bar{M}|+1}{b}}) = 1+\log_2(\frac{\sum_{b=0}^{N-K}\binom{N-|\bar{M}|}{b})}{\sum_{b=0}^{N-K}\binom{N-|\bar{M}|+1}{b}}) = 1-\log_2(\frac{\sum_{b=0}^{N-K}\binom{N-|\bar{M}|+1}{b}}{\sum_{b=0}^{N-K}\binom{N-|\bar{M}|}{b})}).$$

This shows the information gain of 1 is only achievable for the special case of $K = N$, which is the trivial case of no intra-order trade-off. Furthermore, since the ratio inside log is decreasing with $N - |\bar{M}|$, the information gain increases as we cut more lateral connections. Same results hold for $\log_2(\frac{\bar{\pi}(|\bar{M}|)}{\bar{\pi}(|\bar{M}|-1)})$ as well.

This helps us to narrow down the scope of our search for the MIP. Consider the cut where one mechanism unit is removed from all the K purview units. This cut removes the minimum number of connections among all the valid cuts. It removes K connections, 1 self connection and $K - 1$ lateral connections. Any other valid cut that removes at least one self connection can only increase the average, compared to this cut. This is because, as shown above, cutting more self connections can only increase the average, except the special case of $K = N$, and cutting more lateral connections from a purview unit also only increases the average. Therefore, the special cut discussed above has smaller average information gain, compared to any other cut that removes at least one self connection. This narrows our search to only this cut and the cuts that do not remove self connections.

We can further show that among the partitions that do not cut self connections bi-partitions have the smallest average information gain. To see this, starting from any partition with more than two parts, merging the two smallest parts decreases the average. This is because the purview units in the smallest parts have the maximum number of connections removed from them. As already shown, the information gain increases as we cut more lateral connections, therefore by merging the smallest parts we can avoid removing the connections with maximum information gain, which can only decrease the average. This narrows the candidate partitions to $\frac{K}{2} + 1$ partitions, *i.e.,* linear growth with size, and makes it computationally feasible to evaluate for bigger networks.

4 Relations' integrated information

In this section, we provide the solution to the optimization problem in (13):

$$\begin{aligned} \max \sum_{i=1}^{|\mathcal{Z}(o)|} \frac{\varphi_{(i)}}{|z_{(i)}|}(2^{|\mathcal{Z}(o)|-i}-1), \\ \text{subject to} \sum_{i=1}^{|\mathcal{Z}(o)|} \frac{\varphi_{(i)}}{|z_{(i)}|} \leq S(o) \end{aligned} \tag{13 repeated}$$

$\frac{\varphi_{(i)}}{|z_{(i)}|}$ is sorted indexing of the elements in $\mathcal{Z}(o)$, such that $(z_{(1)}, \varphi_{(1)})$ has the smallest $\frac{\varphi}{|z|}$ ratio, $(z_{(2)}, \varphi_{(2)})$ has the second smallest $\frac{\varphi}{|z|}$ ratio, and so on. Thus, we need to formalize the constraint that $\frac{\varphi_{(i)}}{|z_{(i)}|}$ is non-decreasing and non-negative by a change of variable as follows:

$$\frac{\varphi(1)}{|z_{(1)}|} = x_1, \frac{\varphi(2)}{|z_{(2)}|} = x_1 + x_2, \ldots, \frac{\varphi(i)}{|z_{(i)}|} = \sum_{j=1}^{i} x_j, \quad x_j \geq 0, \forall j$$

In other words, we are defining $\frac{\varphi_{(i)}}{|z_{(i)}|} = \frac{\varphi_{(i-1)}}{|z_{(i-1)}|} + x_i$, where x_i is a non-negative value. This translates the problem in (13) into:

$$\begin{aligned} \max \sum_{i=1}^{|\mathcal{Z}(o)|} (2^{|\mathcal{Z}(o)|-i}-1) \sum_{j=1}^{i} x_j, \\ \text{subject to} \sum_{i=1}^{|\mathcal{Z}(o)|} \sum_{j=1}^{i} x_j = \sum_{i=1}^{|\mathcal{Z}(o)|} (|\mathcal{Z}(o)| - i + 1) x_i \leq S(o) \\ x_i \geq 0, \ i = 1, \ldots, |\mathcal{Z}(o)|. \end{aligned} \tag{22}$$

This is a relatively easy problem to solve, as it includes maximizing a linear function of $|\mathcal{Z}(o)|$ variables, given $|\mathcal{Z}(o)|+1$ linear constraints. The solution to the problem is one of the $|\mathcal{Z}(o)|+1$ vertices defined by the constraints. The first vertex is $x_i = 0, \forall i$, which is the trivial minimum of the problem. The rest of the vertices have the same property that for some k, $x_k = \frac{S(o)}{|\mathcal{Z}(o)|-k+1}$ and $x_i = 0, i \neq k$. For such vertex, the value of the objective function is:

$$\frac{S(o)}{|\mathcal{Z}(o)|-k+1} \sum_{i=k}^{|\mathcal{Z}(o)|} (2^{|\mathcal{Z}(o)|-i}-1) = S(o) \left(\frac{2^{|\mathcal{Z}(o)|-k+1}}{|\mathcal{Z}(o)|-k+1} (1 - \frac{1}{2^{|\mathcal{Z}(o)|}}) - 1 \right).$$

This value is monotonically decreasing with k, which means the maximum occurs at the vertex where $x_1 = \frac{S(o)}{|\mathcal{Z}(o)|}$ and $x_i = 0$ for $i = 2, \ldots, |\mathcal{Z}(o)|$. This corresponds to a system where all the distinctions in $\mathcal{Z}(o)$ have the same $\frac{\varphi}{|z|}$ value, i.e. $\frac{\varphi_{(i)}}{|z_{(i)}|} = \frac{S(o)}{|\mathcal{Z}(o)|}, \forall i$. In other words, if we distribute the sum, $S(o)$, such that the distinction integrated information is proportional to its $|z_c^* \cup z_e^*|$ for all the distinctions, we can achieve the maximum value of:

$$S(o)\left(\frac{2^{|\mathcal{Z}(o)|}}{|\mathcal{Z}(o)|}(1 - \frac{1}{2^{|\mathcal{Z}(o)|}}) - 1\right).$$
